# A Proof-of-Concept Study to Develop a Peptide-Based Vaccine against Salmon Lice Infestation in Atlantic Salmon (*Salmo salar* L.)

**DOI:** 10.3390/vaccines12050456

**Published:** 2024-04-24

**Authors:** Amritha Johny, Pedro Ilardi, Rolf Erik Olsen, Bjørg Egelandsdal, Erik Slinde

**Affiliations:** 1 Faculty of Chemistry, Biotechnology and Food Science, Norwegian University of Life Sciences, 1433 Aas, Norway; bjorg.egelandsdal@nmbu.no (B.E.); slinde.erik@gmail.com (E.S.); 2 Farmacologia en Aquacultura Veterinaria FAV S.A., 295, Pedro de Valdivia Avenue, Santiago 7500524, Chile; 3 Department of Biology, Faculty of Natural Sciences, Norwegian University of Science and Technology Sealab, 7010 Trondheim, Norway; rolf.e.olsen@ntnu.no

**Keywords:** *Lepeophtheirus salmonis*, *Caligus rogercresseyi*, fish parasite, blood feeding, peptide epitope, LC-MS/MS

## Abstract

Proteins present in blood samples from Atlantic salmon (*Salmo salar*) infected with salmon lice (*Lepeophtheirus salmonis*) were analyzed using liquid chromatography–high-resolution mass spectrometry. Bioinformatic analyses revealed 1820 proteins, of which 58 were assigned to lice. Among these, peroxiredoxin-2, an antioxidant protein, was found relevant with respect to blood feeding of the parasite. The three-dimensional structure analysis of the protein revealed a surface amino acid sequence of interest. A 13-amino-acid peptide was selected as a potential antigen due to its predicted solubility, antigenicity, probable non-allergenic, and non-toxic nature. This peroxiredoxin-2-derived peptide was synthesized, combined with a commercially available adjuvant, and used for vaccination. The test vaccine demonstrated a 60–70% protection rate against early-stage *Lepeophtheirus salmonis* infection in a challenge trial in Norway. Additionally, the vaccine was tested against salmon lice (*Caligus rogercresseyi*) in Chile, where a remarkable 92% reduction in the number of adult lice was observed. Thus, in combination with the selected adjuvant, the peptide showed antigenic potential, making it a suitable candidate for future vaccine development. The approach described holds promise for the development of peptide vaccines against various ectoparasites feeding on blood or skin secretions of their hosts.

## 1. Introduction

Salmon lice represent a large economic burden for fish farmers. The lice are obligate ectoparasitic copepods on the external surface of marine fish. The term salmon louse or sea louse commonly refers to *Lepeophtheirus* spp. and *Caligus* spp. These species pose an important parasitic problem in salmonid aquaculture because of their harmful effects on fish growth, disease susceptibility, and survival [1]. The life cycle of *Lepeophtheirus* spp. consists of eight stages: two planktonic nauplii, one infective copepod, two attached chalimus, two mobile pre-adult, and one adult male and female stage [2]. The chalimus stage is of particular interest in vaccine development, because during this stage, lice are assumed to have their first interaction with the host through the development of a frontal filament (FF) and subsequent blood feeding, followed by a transition into the adult stage [3]. Likewise, the life cycle of *Caligus* spp. copepods also includes eight developmental phases. Among these, three are planktonic, while the remaining five are parasitic [4]. The planktonic phases consist of two naupliar stages and one copepodid stage, which is parasitic. The other five parasitic stages include four chalimus stages that attach to the host fish via the FF, as well as the male or female adult stages. The species that significantly impact salmonid aquaculture are *Lepeophtheirus salmonis* in the Northern Hemisphere and *Caligus rogercresseyi* in the Southern Hemisphere [5]. Despite being in the same family, there are differences in the feeding of these ectoparasites. *L. salmonis* obtains nourishment from host blood during its juvenile and adult stages, whereas *C. rogercresseyi* predominantly feeds on the skin mucous of fish hosts [2,6]. Studies about the FF are incomplete, but it is assumed to serve primarily as an attachment structure that re-grows during each molting until the parasite has reached the pre-adult stage. Studies have also pointed to its association with the frontal and lateral glands of the lice, shedding light on a role beyond attachment in *L. salmonis* [3]. The possibility cannot be ruled out that the lice use their FF to secrete chemical compounds such as anti-coagulants, immunosuppressants, or other unknowns to aid in parasitism. Such a mechanism may explain the low or absent response of fish to lice attachment. The rationale of our experiment rests on the possible existence of said type of mechanism.

Epitope-based (synthetic peptide) vaccines are regarded as a new generation of vaccines expected to show larger specificity and fewer side effects. Nevertheless, the success of a vaccine depends mainly on the choice of a suitable epitope. We chose to use an epitope selected from peroxiredoxin-2 of *L. salmonis*, detected in the blood of lice-infected Atlantic salmon (*Salmo salar*), and produced a test vaccine [7]. The efficacy of the vaccine against two louse species, differing in their mode of feeding, was subsequently assessed. Our hypothesis in this proof-of-concept study is that (1) a vaccine made from proteins of louse origin found in Atlantic salmon will induce protection of the salmon, and (2) this protection impacts directly the lice via blood meals, in the host–parasite interface while feeding, or through host skin secretions.

## 2. Materials and Methods

### 2.1. Experimental Facility and Animal Ethics

The vaccine trial in Atlantic salmon was conducted at the Matre Research Station of the Institute of Marine Research, Bergen, in accordance with relevant guidelines and approval granted by the Norwegian Food Safety Authority (FOTS ID: 28713 (21 October 2021); 29599, (27 March 2022)). The vaccine trial in Atlantic salmon in Chile was conducted at CRC Innovación (Puerto Montt, X Región, Chile) under the ethical guidelines.

### 2.2. Screening of Proteins and Selection of Candidate Peptide

Blood samples were collected from Atlantic salmon (n = 6) infested with *L. salmonis,* screened using liquid chromatography–high-resolution mass spectrometry (Q-Exactive mass spectrometer, Thermo Scientific, Waltham, MA, USA), and analyzed using PEAKS Studio version 8.5 from Bioinformatic solutions. The sequences of louse proteins were retrieved from the NCBI database (https://www.ncbi.nlm.nih.gov, accessed on 7 March 2021). The analyses yielded a total of 58 proteins of lice origin in the salmon blood. Individual proteins were thoroughly studied based on their biochemical properties and role in antiparasitic activity, as reported in the literature. Only, a few proteins of high relevance were shortlisted [8,9,10,11]. Protein sequences (with >70% identity match to *L. salmonis*) from the Uniprot database were compared to similar protein sequences from Atlantic salmon and rainbow trout using multiple sequence aligning tools (https://www.ebi.ac.uk/Tools/msa/muscle/, accessed on 7 March 2021), and several short sequences of 10–13 amino acids unique to salmon lice were identified. Finally, peroxiredoxin-2 was selected for further development due to its antioxidant and detoxification properties [12,13,14,15].

Structure predictions of protein sequences were performed using SWISS-MODEL (expasy.org) or Robetta web server (https://robetta.bakerlab.org, accessed on 8 March 2021) based on the availability of references with a matching identity of 60% or more. Hydrophilicity and surface peptide properties are essential for effective emulsification in vaccine production. Therefore, the peptide sequence predicted to be located on the surface of peroxiredoxin-2 was selected as the test candidate for the experimental vaccine.

The selected sequence was further screened for its antigenic propensity using the antigenic peptides prediction tool (VaxiJen 2.0 server) with a prediction accuracy of 70 to 89% (http://www.ddg-pharmfac.net/vaxijen/VaxiJen/, accessed on 11 November 2022). The threshold was set to 0.4, i.e., peptides with a score > 0.4 are considered antigenic [16]. Allergenicity of the protein from lice was predicted as probable non-allergen using AllergenFP V.1.0 and AllerTOP v. 2.0 server (accessed on 11 November 2022). The toxicity of the peptide was predicted using the (https://webs.iiitd.edu.in/raghava/toxinpred/algo.php, accessed on 11 November 2022) database; the selected peptide was predicted as non-toxic at an SVM (support vector machine) threshold of −0.83.

### 2.3. Peptide Synthesis and Vaccine Emulsification

The peptide selected was synthesized in two purity levels (98% and 70%) and supplied as a lyophilized powder by ProImmune (Oxford, UK). The vaccines with 98% and 70% purity peptides were produced by Pharma Production AS (Oslo, Norway) using the adjuvant Montanide™ ISA 763 A VG from Seppic (Courbevoie, France). A water-in-oil emulsion was prepared (70 g adjuvant: 30 g aqueous antigenic medium for 100 g of each vaccine) using a high shear mixer (ULTRA-TURRAX® homogenizer (IKA-T10)) as per the SEPPIC protocol. The emulsion stability was assessed through visual examination prior to use. Injection volume was 1 µg peptide/g fish. The emulsified vaccines were kept in a refrigerator (4 °C) until use.

### 2.4. Experimental Groups and Vaccination

#### 2.4.1. Challenge Trial in Norway

Atlantic salmon smolts (vaccinated against bacterial diseases under routine research station protocol), with an average body weight of 100 ± 5 g, were used in the challenge trial. The experimental design consisted of 400 L seawater tanks with 40 fish per tank and one tank per treatment. Fish were maintained at 13 °C, 25 ppt salinity, and a photoperiod of 12 h light–12 h dark for one week. Prior to vaccination, fish were carefully netted from the tank into a 10 L bucket and anesthetized using Finquel (60 mg/L). Vaccination was performed intra-peritoneally (i.p.) with a dose of 1 µg of peptide/g fish using a 20-gauge needle. Fish were not fed for two days after vaccination. Afterward, automated feeding using commercially available feed was provided ad libitum. One week after the vaccination, salinity was raised to 34 ppt. The water level was maintained at the same level in all tanks throughout the experiment, but the photoperiod was adjusted to 24 h light–0 h dark after the first week. After 455 degree-days (the product of water temperature and time in days), the water volume was reduced to 150 L and water flow was stopped and 800 infective copepods from a wild strain of salmon lice were distributed evenly into all tanks (i.e., 20 copepodites per fish). After 10 min, the water level and water flow were restored. Total lice load and documentation of the life stages of the lice were recorded for all fishes at 10, 17, 24, 41, and 51 days post-infection (dpi). At 51 dpi, all fish were euthanized, and the experiment was terminated.

#### 2.4.2. Challenge Trial in Chile

Post-smolt Atlantic salmon (without any previous vaccinations) with an average weight of 250 ± 5 g were purchased from a commercial farm at Puerto Montt. Fish were maintained at the CRC Innovación facility in 500 L seawater tanks with 45 fish per tank, with a flow rate of 1.5 L/s, at 13 ± 1 °C, and a photoperiod of 12 h light–12 h dark. Fish were fed with a commercial diet through automatic feeders ad libitum and were acclimated for at least two weeks before injection. Water quality indicators, such as dissolved oxygen, pH, nitrite, and ammonia, were periodically analyzed, and all recorded measurements were within the acceptable ranges for *S. salar.* After the acclimatization period of two weeks, the fish were fasted for 24 h before vaccination. Fish were anesthetized with Benzocaine (250 mg/L) and vaccinated with a 70% peptide vaccine by i.p. injection using a SOCOREX^®^ 187 Classic syringe (Socorex ISBA SA, Ecublens, Switzerland) with 23-gauge needles; 1 μg/g peptide/fish. Fish in the control group were injected i.p. with an equal volume of sterile saline. A total of 135 fish were vaccinated with the experimental peptide vaccine and distributed into 3 tanks with 45 fish each; an equal number of fish from the control group was distributed similarly into triplicate tanks. At 637 degree-days post-vaccination, fish were challenged with *C. rogercresseyi* copepodites (25 copepodites per fish). The copepodites used for the challenge experiments were obtained from ovigerous female salmon lice collected during the harvest of Atlantic salmon at a commercial fish farm located in the X Región in Chile. After initial exposure, the copepodite load per Atlantic salmon was determined by performing visual counts on five fish per group at 5 dpi (early infestation) to confirm the attachment of parasites to fish. Subsequently, lice counts were performed at 12 dpi (juvenile stage) and 20 dpi (adult stage) on all fishes.

### 2.5. Statistical Analyses

All the statistical analyses were performed using GraphPad Prism, version 10. Significant differences among groups were assessed by non-parametric tests (Kruskal–Wallis test) followed by Dunn’s post hoc test (α = 0.05) and Bonferroni correction of *p*-values.

## 3. Results

### 3.1. In Silico Analyses

High-throughput proteomic analyses of blood samples from Atlantic salmon with a high lice load yielded 1762 proteins identified from salmon and 58 proteins from salmon lice. Peroxiredoxin-2 was selected due to its importance in the blood-feeding stage of the parasite. A short peptide of 13 amino acids (NKEFKEVSLKDYT) unique to salmon lice was selected for vaccine construction. The predicted three-dimensional structure of peroxiredoxin-2 confirmed that the selected peptide sequence was a surface peptide, and it was hypothesized to have accessibility to the immune system of the fish as well as other binding ligands. The synthesized peptides (two purity levels) could easily be emulsified confirming their hydrophilic–lipophilic balance.

### 3.2. Vaccination

A dose of 1 µg/g (peptide/fish) was selected for vaccination based on previous vaccine trials conducted by the same group of authors and on available literature [17,18]. No fish mortality, pigmentation, or deposition of vaccine residues around the vaccinated area in fish were observed after injection with the experimental peptide vaccines towards *L. salmonis* or *C. rogercresseyi* when compared with the controls.

#### 3.2.1. Challenge Experiment in Norway in Atlantic Salmon with *L. salmonis*

The behavior of lice depends on the health status of individual fish, the flow of water, temperature, as well as other factors. Thus, tank experiments are in general difficult to conduct, and results are difficult to be extrapolated as predictions for field experiments. Lice moving from one host to another would cause inaccurate data.

**Figure 1:**
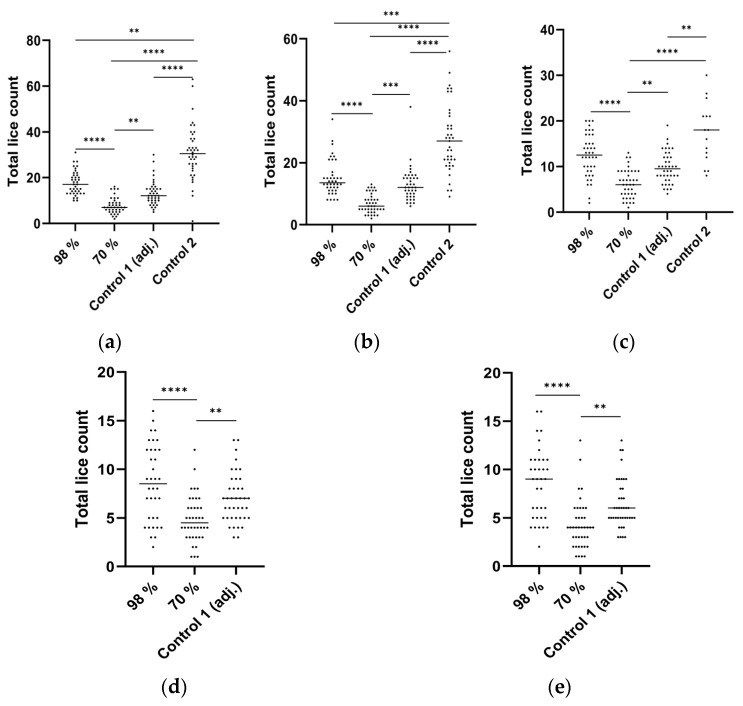
Total lice count on individual fishes (n = 40), vaccinated with 98% peroxiredoxin-2, 70% peroxiredoxin-2, with adjuvant (Control 1), and without vaccination/untreated (Control 2). Scatter plots are of lice counts recorded on days post-infection, at 5 dpi (**a**), 17 dpi (**b**), 24 dpi (**c**), 42 dpi (**d**), and 51 dpi (**e**) in Atlantic salmon after *L. salmonis* challenge in Norway. Fish in the Control 2 group were euthanized at 24 dpi due to ulcer development and poor welfare. Each dot represents one fish. Horizontal lines in the plots represent the median lice counts in respective groups. Asterisks denote significant differences (**** *p* ≤ 0.0001; *** *p* ≤ 0.001; ** *p* ≤ 0.01, and *p* > 0.05 is non-significant and not shown).

In general, we observed a continuous loss of lice throughout the experiment, as shown in Figure 1. However, challenge experiments with copepods gave an initial attachment of approximately 20 copepodites per fish as calculated (Figure 1a), which shows the efficiency of the model. Adjuvant and the 70% pure peptide led to a significant reduction in lice when compared to control groups (Figure 1a–c), with almost 70% efficacy at 24 dpi when compared to the untreated control group (Control 2) (Figure 1c). However, no significant difference was observed between the 98% purity peptide and the Control 1 group after 24 dpi (Figure 1c). The reason for the difference in protection observed for 98% and 70% purified vaccine is unknown. Control with adjuvant (Control 1) also may have stimulated the immune system, resulting in a difference in lice counts compared to the untreated control group (Control 2). Mortality was lower in all vaccinated fish in this trial when compared to the untreated control group (Control 2). This was evident from the fact that only the fish in the untreated control group (Control 2) had to be euthanized at 24 dpi due to large ulcers developed on the body surface, resembling bacterial infection rather than being caused by lice. In general, peptide vaccines have the potential to stimulate cellular immunity [9]; however, further investigation is required to assess the effectiveness of the selected peptide in triggering humoral responses.

#### 3.2.2. Challenge Experiment in Chile in Atlantic Salmon with *C. rogercresseyi*

**Figure 2:**
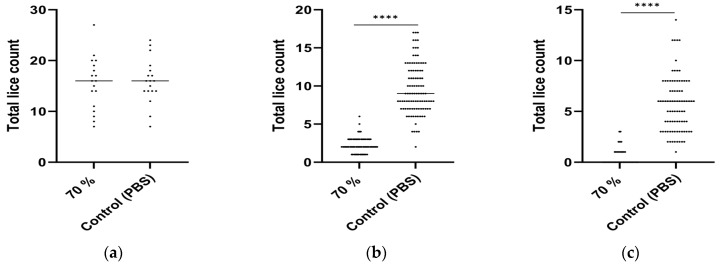
Total lice count on individual fishes vaccinated with 70% peroxiredoxin-2 peptide and the control with phosphate-buffered saline, PBS. Scatter plots are of lice counts recorded on early infestation (702 degree-days or 5 dpi) (**a**), juvenile louse stage (793 degree-days or 12 dpi) (**b**), adult louse stage (897 degree-days or 20 dpi) (**c**) in Atlantic salmon after *C. rogercresseyi* challenge in Chile. The number of individual fish counted in the 70% group and control group is 18 each at 5 dpi, 101 and 100 at 12 dpi, and 90 and 85 at 20 dpi. Each dot represents one fish. Horizontal lines in the plots represent the median lice counts in respective groups. Asterisks denote significant differences (**** *p* ≤ 0.0001; *p* > 0.05 is non-significant and not shown).

The effectiveness of the peptide vaccine (70% purity) developed for *L. salmonis* was also tested against *C. rogercresseyi*, the species of salmon lice in the southern hemisphere. Figure 2 illustrates the vaccine’s impact on total lice load. The average infestation of copepods at 5 dpi was comparable between vaccinated and control groups (15.5 ± 5.2 and 15.9 ± 4.4, respectively), indicating the effectiveness of attachment in the model (Figure 2a). During subsequent days, a significant reduction in the total *Caligus* load was observed in the vaccinated group during both juvenile (12 dpi) and adult (20 dpi) stages, with the latter stage showing a remarkable reduction of 92%.

## 4. Discussion

We have analyzed the blood of salmon heavily infected with salmon lice, *L. salmonis,* and identified 58 proteins of louse origin. After evaluating the biochemical and physiological role of these proteins, peroxiredoxin-2 was selected for this study based on its antioxidant properties, which are important during the parasite’s blood feeding. Production of a recombinant peroxiredoxin-2-based vaccine for fish was found too expensive; therefore, the synthetic peptide approach was tested. A 13-amino-acid sequence on the protein surface was selected as an antigen and tested at two purity levels. In silico analyses suggested the peptide to be antigenic, water-soluble, and non-toxic. The challenge study indicates that the vaccines produced from the peptide derived from peroxiredoxin-2 can function as an antigen and provide protection against both species of salmon lice, particularly *C. rogercresseyi*. However, the use of a peptide vaccine may have a low success rate. Proteolysis may occur in the fish body, and there is uncertainty as to possible antibody breakdown by lice proteases. In our experiments, we found that a dose of 1 µg peptide of 70% purity/g of fish gave a reasonable reduction in end lice count. Previous studies in this area, which gave an increased humoral response, were not very successful in reducing parasitic load [19]. Therefore, in this study, reduction in lice load was prioritized [20] and recorded as the key parameter.

Salmon lice, like ticks, are hematophagous arthropods that require blood feeding for nutrition and transition into the next growth stage [21]. However, disruption of their antioxidant system may lead to a high level of oxidative free radicals, which in turn causes oxidative stress and cytotoxicity [22,23,24]. In a previous study in ticks, antioxidant enzymes like catalases and peroxiredoxins were found to be present, and gene silencing of peroxiredoxins affected blood feeding and oviposition; however, gene silencing of catalase did not show any significant effect [13,25,26,27]. These results indicate that peroxiredoxins may have an important role in tick blood feeding and oviposition. Furthermore, peroxiredoxins are also considered to be vaccine candidates for other parasitic species, like *Leishmania donovani* [28] and *Fasciola hepatica* [29]. At the infective stage, *F*. *hepatica* excysts from a dormant state, penetrates the intestinal wall, and migrates to the liver. They secrete peroxiredoxin into their host to regulate their environment for survival [30,31]. Thus, peroxiredoxins can be a potential target for parasite control by exploiting oxidative stress coping mechanisms during the blood-feeding parasitic stages. Also, in endoparasites, peroxiredoxins have been shown to be the most important detoxifying enzymes for parasite survival [31,32], making them suitable vaccine candidates [33,34]. These proteins may not be the only ones responsible for detoxifying action or inhibition of the host defense system, but depending on the particular stage in the life cycle and mode of feeding of the parasites, may play a supportive role in maintaining the redox balance. This may be one reason for not observing any reduction in lice count in the *L. salmonis* challenge trial, which could play a major role against *C. rogercresseyi* (Figure 1c vs. Figure 2c).

It is of interest to note that the less purified peptide gave better protection than the more purified one against *L. salmonis*. The possibility of impurities interfering with the biological function in a peptide vaccine cannot be ruled out; future studies using analytical techniques to confirm the homogeneity, stability, identity, and purity of peptide in the emulsified vaccine must be performed [35]. Among the two controls in the *L. salmonis* challenge, the group with adjuvant injected (Control 1) suggested a general stimulation of the immune system, which in turn provided limited protection towards the challenge with copepodites when compared to control without adjuvant (Control 2). As a next step, different adjuvants will be tested to understand their interactions with components of the immune system of the fish and their efficacy in reducing lice loads.

Although *L. salmonis* and *C. rogercresseyi* have different modes of feeding, indications on the facultative hematophagous nature of the female adults of *C. rogercresseyi* along with similarities in heme biosynthesis and the antioxidant system in both species [36] point to a common mechanism in the feeding process, which led us to test the selected peptide in our pilot study. The reduction in the lice count in vaccinated fish supports our hypothesis. However, the higher reduction in the load of *C. rogercresseyi* upon vaccination also raises questions about modulators of host resistance within the mucus, as mentioned by Fast et al. [37]. Moreover, the ability of the antigenic protein to induce the production of specific antibodies in salmon mucus cannot be ruled out [18,38]. Further research is warranted considering the potential of antioxidant systems in parasitic defense for vaccine development [39,40,41]. This proof-of-concept study contributes to the limited body of research focusing on antioxidant systems in salmon lice and also shows the possibility of developing a vaccine based on proteins of louse origin detected in Atlantic salmon blood, suggesting potential avenues for vaccine development.

## 5. Conclusions

This study demonstrates the possibility of developing a cost-effective synthetic peptide vaccine based on the secretions injected into the host by the parasite. The louse protein, peroxiredoxin-2, selected from the blood of lice-infested Atlantic salmon is assumed to have a role in antioxidative mechanisms facilitating the feeding process of the parasite. On vaccination of Atlantic salmon with experimental vaccine (70% purity peptide), better protection was achieved against *C. rogercresseyi* when compared to *L. salmonis* in Atlantic salmon. Further studies are required to comprehensively document relevant immunological parameters, understand the difference in mechanism of action of the candidate peptide in both species of louse, and validate these findings. Insights from these findings could also have implications for other blood-feeding external parasites. Vaccines alone, or in combination with other approaches, e.g., antiparasitic feed, or genetic interventions, can alleviate the aquaculture industry’s dependence on stressful delousing treatments. Such alternative strategies, which could be effective, safe, and pose minimal health and welfare risks to fish, are highly needed. This could ultimately enhance the overall health of the fish, enabling them to better combat infections, thereby addressing a longstanding issue in salmonid farming.

## Data Availability

The original contributions presented in the study are included in the article, further inquiries can be directed to the corresponding author.
